# When Covid‐19 meets conflict: politics of the pandemic response in fragile and conflict‐affected states

**DOI:** 10.1111/disa.12514

**Published:** 2021-11-11

**Authors:** Dorothea Hilhorst, Rodrigo Mena

**Affiliations:** ^1^ Professor, International Institute of Social Studies Erasmus University Rotterdam The Netherlands; ^2^ Assistant Professor, International Institute of Social Studies Erasmus University Rotterdam The Netherlands

**Keywords:** conflict, Covid‐19, disaster governance, disaster risk creation, politics of disasters, vulnerability

## Abstract

The Covid‐19 pandemic has magnified existing crises and vulnerabilities, but much remains unknown about how it has affected fragile and conflict‐affected settings. This paper builds on the theory that hazards become a disaster in interaction with vulnerability and response policies, yet often lead to renewed disaster risk creation. It is based on seven case studies of countries worldwide that experienced social conflict at the advent of the pandemic, covering the period from March–August 2020. The findings show that authorities instrumentalised Covid‐19 to strengthen their control and agendas. Responsibility was assumed for lockdowns, but this was not accompanied by care to mitigate their adverse effects. Social conflict shaped the response, as high levels of mistrust in authorities complicated the implementation of measures, while authorities did not support community‐based coping initiatives. Whether Covid‐19 will trigger or exacerbate conflict and vulnerabilities depends on pre‐existing, country‐specific conditions, and how a government and other actors frame the issue and respond.

## Introduction

As Covid‐19 swept the globe in 2020, how low‐income countries and vulnerable populations would be affected and able to respond soon became matters of concern (International Crisis Group, 2020; Mena, 2020). Similarly, it was queried how the pandemic would exacerbate inequalities, and compound problems already prevalent in those areas where humanitarian crises or conflict were ongoing (Desportes, 2020; Hilhorst, 2020). In response, the authors teamed up with students at the International Institute of Social Studies (ISS) in The Netherlands to study the effects of Covid‐19 and the measures taken in seven countries, for six months from March–August 2020. Seven couples were formed with researchers from or working in those countries, who analysed international and national news outlets and reports, and complemented their findings with online key informant interviews. The results from the cases, detailed below, were published in blog posts, working papers, and reports. This paper provides a qualitative meta‐analysis of those results to understand the politics of Covid‐19 responses better. This means that we have undertaken a thematic secondary qualitative analysis of the primary findings of the country studies.

The investigation into Covid‐19 responses was part of the final phase of a five‐year research programme on cases where disasters meet conflict. This programme centred on three major themes:

- the relevance of critical disaster studies that seek to distinguish hazards from vulnerability and response capacities, emphasising that disasters are never ‘natural’;
- the interaction of crises when they are compounded, that is, how the dynamics of conflict and disaster affect each other; and
- how conflict magnifies the politics of disaster.

In a similar vein, we set out to unravel how the hazard of Covid‐19 led (or not) to disaster, how the pandemic interacted with enduring crises, and if and how governments or other actors instrumentalised measures surrounding the outbreak to advance their interests and politics. In addition, we paid special attention to the tension between top‐down and bottom‐up responses to Covid‐19, as this was a recurring theme worldwide. There is no doubt that top‐down policies and expert knowledge are required to curb a pandemic, but there were many controversies around this from the beginning, leading to social protest about how governments imposed far‐reaching regulations, while overlooking the potential of community‐based responses. We expected this to be especially pronounced in conflict‐affected areas that were already characterised by high levels of distrust in state authorities.

The study covered seven countries that have been affected by conflict in different ways. The first group of countries was composed of low‐intensity or post‐conflict societies known to have fragile institutions. This group constituted the Democratic Republic of the Congo (DRC), Haiti, and Zimbabwe. In these countries, the state is seen to be centralised, in some ways fragile, and in other ways highly organised, such as concerning surveillance. The state is seen to be far removed from the communities, and although there are authority structures at the local level, people frequently strongly mistrust them. The second group of countries was composed of strong or authoritarian states, with a high prevalence of social conflict and a vocal civil society and other institutions with countervailing powers that challenge the states' actions. This group constituted Brazil, Chile, India, and the Philippines. A potential third group of high‐intensity conflict areas, comprising, for instance, Afghanistan and Yemen, was not included for several reasons, including a lack of access to data and non‐availability of national researchers at the ISS.

The paper next reviews the emerging literature on Covid‐19 and conflict and elaborates on the research themes, before presenting the methodology, the cases, and the meta‐analysis of the country studies.

## Covid‐19 as a disaster

Covid‐19 may be seen as subject to the ‘iron law’ of disaster studies that a disaster cannot be equated to the hazard (Wisner, Gaillard, and Kelman, 2012). Disaster risks result from hazards encountering vulnerability, mitigated by response capacities (Wisner et al., 2004). Whether a disaster unfolds upon an earthquake, for example, depends on the level of prevention in the built environment in combination with poverty levels among the population (Kelman, 2020a; Wisner, Gaillard, and Kelman, 2012). While Covid‐19 has been prominent worldwide and has had significant effects on populations in high‐income countries, there is ample evidence that marginalised and vulnerable populations are at greater risk of becoming infected (Raju and Ayeb‐Karlsson, 2020).

In the case of Covid‐19, global preparedness was wanting, despite long‐time warnings that risks of pandemics were increasing, due to ‘intensification of international travel, trade and livestock husbandry, as well as increasing human population density and changing interactions between humans and wild animals’ (Oppenheim et al., 2019, p. 1). The Epidemic Preparedness Index and the Infectious Disease Vulnerability Index showed that vast parts of the world were unprepared to manage a large‐scale infectious disease epidemic (Moore et al., 2016), despite consistent warnings following previous events, such as Spanish Flu, HIV/AIDS, SARS, and Ebola (Bergeijk, 2021). The lack of preparedness may be seen as a major contributor to the disastrous impact of the virus, and conflict‐affected countries were among the least prepared for Covid‐19 (International Crisis Group, 2020; Mustasilta, 2020).

Responses to disaster are designed to prevent or reduce risk, but they can also lead to further disaster risk creation (Lewis and Kelman, 2012; Wisner and Lavell, 2017). Covid‐19 is a hazard turned into a disaster owing to the actions taken (or not) to prevent and respond to it (Hilhorst, 2020; Kelman, 2020a, 2020b, p. 297). Covid‐19 did not by itself produce the risks that we have seen unfold throughout the world, and major research questions have become pertinent about the impact of response policies: whether they have been effective, resulted in a net increase or decrease of risks, their unintended consequences, and the possible wicked side‐effects that may lead to greater risk of infection. Viewing Covid‐19 as a disaster thus compels us to ask how the risk evolves in the interplay of hazard and response policy, and how risks are allocated and affected by vulnerability.

**Figure 1:**
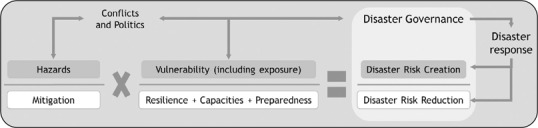
Disaster risk creation and risk reduction **Source**: authors.

The ‘logic’ of disasters discussed is summarised in Figure 1, which schematically represents how disasters emerge and could be reduced.

## Disaster politics of Covid‐19

It is accepted wisdom in disaster studies that disasters are political events, yet this notion is difficult to secure in public and popular thinking about disasters. As detailed above, disaster risks are largely human‐created and hence subject to the politics of prioritising (economic) interests over reducing them, foregrounding certain risks while ignoring others. Similarly, once disasters happen, the response becomes political within seconds of their occurrence (Olson, 2000). Of particular importance are the politics of securitisation and instrumentalisation. Securitisation refers to a political ‘speech act’, that is, a publicly accepted declaration of a vital threat that requires extraordinary actions (Waever, 1993; Buzan, Waever, and de Wilde, 1998). It lends a sense of urgency and priority to a particular threat and therefore creates a ‘state of exception’. Successful securitisation results in a political situation where first, all other concerns are overtaken by a single threat. Second, this is used to justify extraordinary measures beyond the established and known rules and politics. Third, where these measures are represented as ‘objectively’ necessary, setting aside the basic tenet of democracy, namely that there are always alternative options that should be subject to political choice (Waever, 1993; Buzan, Waever, and de Wilde, 1998).

A certain securitisation may be seen as a proper response to an emergency like Covid‐19. However, it is often subject to politics of instrumentalisation, where measures presented as crisis response have other or secondary political objectives, such as tightening control, curbing civil society's freedoms, silencing the media, or undermining political opponents.

Instrumentalisation can take the form of *over*‐securitisation when risks are presented as existential threats, as well as *under*‐securitisation (or *de*‐securitisation) (Waever, 1993) when risks are denied or belittled for political purposes. Authoritarian regimes, for example, are notorious for underreporting disaster impacts to retain their image of control (Desportes and Hilhorst, 2020). In the case of Covid‐19, political speak is seen to bring out nationalism (such as in vaccination politics) and the externalisation of the responsibilities of the negative consequences of the pandemic (Hoffmann Pfrimer and Barbosa, Jr., 2020). Moreover, instrumentalisation and political exploitation of the pandemic can lead to violation of human rights by imposing severe sanctions on civilians (Nygård, Methi, and Rustad, 2020). Studying disaster politics thus implies a focus on the ways in which disasters are framed, as securitisation and instrumentalisation come with particular interpretations of the nature of the crisis, its causes (and who is to blame), and solutions.

## Top‐down approaches in disaster governance

Disaster response used to entail strict top‐down, command‐and‐control measures geared towards bringing order to the ‘chaos’ and a return to ‘normal’. This has been overtaken by insights that an inclusive governance model is more effective, especially when taking into account disaster risk reduction and long‐term recovery. The current Sendai Framework for Disaster Risk Reduction (2015–2030) builds on an inclusive governance model for disaster response and disaster risk reduction (Djalante and Lassa, 2019). The pace, commitment, and extent to which the consensus on this model is translated in practice vary considerably across the world. Still, there have been significant moves towards governance models that bring together different state and non‐state actors and combine top‐down and bottom‐up approaches (Hilhorst, Boersma, and Raju, 2020). With regard to pandemics, there have been similar calls to complement top‐down state‐centred control with more inclusive and collaborative forms of governance. The Ebola crisis in Sierra Leone in 2014 is a clear case in point, where the reluctance to involve traditional leaders and community health workers in the response proved to be very counter‐productive (Mallett and Denney, 2015; Parker et al., 2019). And in Syria, for example, bottom‐up responses and the role of local volunteers and leaders were the foundation for scaling up regional response to Covid‐19 (Ekzayez et al., 2020). Ignoring, or even stifling, instead of building on community‐based responses to disaster is especially ineffective where states are fragile or limited in their capacity to deal with the impact of the disaster and the consequences of the response policies.

## The nexus between Covid‐19 and conflict

The nexus between Covid‐19 and conflict concerns, first, the effects of the pandemic on the intensity and dynamics of conflict, and, second, the effects of conflict on the impact of and responses to the pandemic.

### Effects of the pandemic on conflict

When it became clear that the ‘global outbreak has the potential to wreak havoc in fragile states, trigger widespread unrest, and severely test international crisis management systems’ (International Crisis Group, 2020, p. 1), multiple actors called for ceasefires and extra efforts to stop conflict to facilitate responses to Covid‐19 (Mehrl and Thurner, 2020; United Nations, 2020a). In April 2020, United Nations (UN) Secretary‐General António Guterres published an ‘Appeal for Global Ceasefire’, indicating the implications of Covid‐19 for international peace and security, and for ground‐level responses and actions in response to the pandemic (United Nations, 2020b). More than 170 UN Member States and several non‐state actors, including armed groups, had endorsed the appeal as of June 2020.

Nonetheless, emerging literature seems to point to a rise in conflict during the Covid‐19 pandemic, even though it is admittedly complicated to establish this correlation. Using the Armed Conflict Location and Event Data Project, Polo (2020) and Bloem and Salemi (2021) found that while some forms of conflict decreased at the beginning of the crisis, such as protests and riots, most countries experiencing violent conflict before the pandemic saw an increment in conflict‐related events during Covid‐19 in comparison to pre‐pandemic levels. Mehrl and Thurner (2020) discovered that while lockdown policies increased armed conflict in the Middle East, the number of violent clashes decreased during Covid‐19 lockdowns in Southeast Asia and the Caucasus. However, they warned that these results might be skewed because of reporting difficulties during Covid‐19. In Afghanistan, the number of violent attacks on civilians has risen since the pandemic (Polo, 2020), many of which have been claimed by the Taliban which is ‘using the coronavirus crisis for propaganda’ (Jackson, 2020). Ide (2021) reached similar conclusions based on a study of nine countries. In four of these (Afghanistan, Colombia, Thailand, and Yemen), armed conflict initially declined, which may be related to a decision by armed groups to use the outbreak as a window of opportunity to gain support and resolve logistical problems in carrying out their activities. In the other five (India, Iraq, Libya, Pakistan, and the Philippines), conflict prevalence increased, in part and importantly due to ‘[t]he weakening of state institutions (providing incentives for rebels to intensify military pressure) and a lack of (international) public attention (allowing to extend military operations without backlashes)’ (Ide, 2021, p. 5).

One reason why conflict may have intensified is related to the global nature of the pandemic. Many countries become more inward‐looking, affecting diplomatic initiatives and distracting or delaying efforts related to peace operations, peace‐building, and conflict resolution mechanisms (International Crisis Group, 2020; Mustasilta, 2020).

The politics of disaster outlined above are also present in the case of conflict. Disasters reveal and affect the social struggles and inherent inequities between actors (Cuny, 1983; Pelling and Dill, 2006; Venugopal and Yasir, 2017; Desportes, 2020). The processes of disaster politics in conflict‐affected areas are not different from disaster situations in more peaceful areas, but they may be magnified and set in sharper relief in view of their relation with life and death. The health‐related, economic, and political consequences of Covid‐19 can thus be seen to add to—or alter—continuing dynamics of conflict and social tensions while being woven into ongoing narratives of conflict. The pandemic can lead to competition over scarce resources, exacerbate root causes of conflict, and be instrumentalised by actors to gain political advantage, territorial control, and advance personal agendas (Brown and Blanc, 2020; Mehrl and Thurner, 2020; Mustasilta, 2020; Polo, 2020; Bloem and Salemi, 2021).

The ‘Armed groups responses to the Covid‐19 crisis’ webinar (ODI, 2020) revealed that instrumentalisation might also be beneficial to the response to Covid‐19. Participants in the live event testified how different armed groups across the world have used the pandemic as an opportunity to further their agendas vis‐à‐vis violent acts, but also as a means to gain legitimacy and social approval. For instance, ‘[t]he Islamic State issued directions on handwashing, and videos emerged of the Taliban enforcing temperature checks. The National Liberation Army (ELN) in Colombia announced lockdown measures, and Hezbollah mobilised thousands of medical personnel. In addition, organised criminal groups in Mexico, Brazil and El Salvador delivered aid packages and enforced curfews to curtail the spread of the virus’ (Jackson and Weigand, 2020). While these activities may be somewhat instrumental to the interests of these groups, they can also be seen as a demonstration of solidarity and good intentions, resulting not only in a reduction in Covid risks, but also possibly in grievances and conflict in the medium or long term (Ide, 2021).

### Compounding effects of conflict and Covid‐19

It is nearly impossible to establish the death toll of Covid‐19 in relation to conflict and fragility. The cases in this paper display large diversity, with an accumulated death toll of 1,089 in the DRC, 657 in Haiti, 4,658 in Zimbabwe, 452,321 in India, 40,675 in the Philippines, 37,609 in Chile, and 603,324 in Brazil, according to numbers (as of 19 October 2021) provided by John Hopkins University (Statista, 2021). The databases rely on governments' statistical reports, and apart from problems of incomparable datasets, data may be incomplete. Other effects, including ailing health, malnutrition, loss of livelihoods, or collapse of services, are even more difficult to determine or have still to be compiled. The World Food Programme, for example, anticipated in June 2020, at the launch of *The State of Food Security and Nutrition in the World*, that an additional 83–132 million people would become malnourished because of the pandemic (WHO, 2020), but actual figures have not yet been given. However, a number of interactions between conflict and Covid‐19 are becoming visible through case‐study evidence that are in line with patterns found at the nexus of disaster and conflict.

The way in which conflict can affect the spread of Covid‐19 varies. Posen (2020) found that conflict is associated with conditions that are beneficial for the spread of diseases, including lower hygienic circumstances and increased poverty levels. Conflict may further contribute to the collapse of local economies (International Crisis Group, 2020). Covid‐19 may compound and exacerbate economic and health crises related to a conflict, as confirmed in a study in Colombia, Libya, Sudan, Ukraine, and Yemen (Mustasilta, 2020), and its spread may be heightened in the densely populated settings of refugee camps (Mena, 2020; Raju and Ayeb‐Karlsson, 2020). Weak health systems and a lack of basic infrastructure are also factors contributing to a rise in the risk posed by Covid‐19 in conflict‐affected locations (Ekzayez et al., 2020). Many of the countries most vulnerable to infectious disease outbreaks, according to the Infectious Disease Vulnerability Index, are affected by violent conflict, including Afghanistan, the DRC, Haiti, Sierra Leone, Somalia, South Sudan, and Yemen (Moore et al., 2016). However, some of the aspects associated with vulnerability to Covid‐19 in non‐conflict and highly developed countries, including an ageing population and intense travelling, may be less applicable in conflict zones, which usually have a demographic youth bulge and where travel is more restricted.

Conflict can further intensify the effects of Covid‐19 by negatively affecting the ability to respond. Conflict produces civilian displacement, low levels of citizen trust in leadership, and fragmented political authority, creating a challenging environment for the implementation of state responses to the pandemic (Brown and Blanc, 2020), as shown, for example, by the case of Mali (Sandnes, 2020). Conflict is also associated with corruption, international sanctions, and high levels of state fragility (Brown and Blanc, 2020; International Crisis Group, 2020), and it limits operational spaces to respond, as observed in Iraq and Somalia, inter alia (Hamasaeed, 2020; Hasan, 2020).

In addition, the resources destined for relief in conflict‐affected areas must now be shared with this new crisis. As stated by the President of the International Committee of the Red Cross and Red Crescent (ICRC), Peter Maurer (2020), ‘[o]ur double response to conflict and Covid‐19 is extra difficult because of the vital measures taken to contain the pandemic’. This situation may be aggravated by the fact that many donor countries are absorbed with their own Covid‐19 crisis. Total humanitarian flows to the DRC and Zimbabwe, for instance, decreased in 2020 as compared to 2019. In Haiti, the humanitarian contribution increased, but only reached 33 per cent of the appeal target. A Covid‐specific appeal in 2020 for these countries only attained 43 per cent for the DRC, 42 per cent for Zimbabwe, and 20 per cent for Haiti (UN OCHA, 2021). In a similar vein, Covid‐19 can lead to a reduction in remittances, as found in Somalia (Blanc, 2020; Elder, 2020; Majid et al., 2020). International vaccination politics and conflict conditions are expected to impact severely on the prospects for vaccination in conflict‐affected areas (Glinski, 2021; Tiller, Devidal, and van Solinge, 2021).

Lastly, emerging scholarship on Covid‐19 and conflict emphasises that, as in any disaster, marginalised and vulnerable populations are at greater risk and suffer more the effects (Raju and Ayeb‐Karlsson, 2020). Covid‐19 augments the vulnerability of people already facing life under conflict in poverty and with a lack of (health) services (International Crisis Group, 2020). For example, in Yemen, the virus arrived in a country already experiencing severe food insecurity and a cholera outbreak (Nagi, 2020). Covid‐19 also unveils vulnerabilities already present in the places affected, explaining the impacts of the pandemic and the need to understand the compound nature of vulnerability and crises (Mena, 2020; Talla Cornejo and Fischer, 2020).

## Methodology

This paper uses the lens of intersecting and compounded crises to analyse local responses to Covid‐19 in seven countries and then the politics of the Covid‐19 response in fragile and conflict‐affected states. We chose a small‐N approach, where a relatively low number of cases enables us to assess qualitatively findings in their context, yet allows for generalisation of the results (Flyvbjerg, 2001). The When Disaster Meets Conflict programme of the ISS worked in three categories of countries:

- high‐intensity conflict;
- low‐intensity conflict in states with authoritarian tendencies; and
- fragile post‐conflict societies.

High‐intensity conflict cases were not included owing, inter alia, to a lack of access to data; hence we used the two other categories of countries. To recap, the first group was composed of four countries with high levels of ongoing social conflict, strong or authoritarian states, and social counter‐movements: Brazil; Chile; India; and the Philippines. The second category was composed of three conflict‐affected areas with fragile governments and institutions: the DRC; Haiti; and Zimbabwe. The exact choice of countries was informed by the availability of national researchers, who had a sound domestic knowledge base, could review secondary sources, and could conduct interviews in their own language.

The case studies were implemented by Master's students and Doctor of Philosophy (PhD) researchers at the ISS, on the basis of secondary sources, and remote interviews were conducted between April and August 2020. The results have been published in multiple blog posts and a series of working papers and reports, as detailed below in the description of the cases. This paper is based on a meta‐analysis of those results, using thematic content analysis.

The analysis focused on four themes: vulnerabilities and their relationship with disaster risk creation and disaster (Covid‐19) impacts; instrumentalisation and securitisation of Covid‐19 responses; top‐down and locally‐led responses to Covid‐19; and the effect and relationship between the Covid‐19 disaster and social conflict. These themes were identified at the start of the research, based on our theoretical approach to disaster in conflict, and initial reading about Covid‐19 in the nations under review and other conflict‐affected areas. They informed initial data gathering in the seven countries, and the different studies were guided by the authors to ensure that they would yield relevant data.

After completion of the country papers, the authors analysed the findings of each of the assessments according to these themes. In addition, a literature review on the topic was conducted that was guided by the same themes, consisting of books, journal articles, reports, and news articles. Given recent developments on the subject of study, and the importance of capturing information on a wide range of actions and responses, the evaluation also included grey literature and audio‐visual material, such as blog entries, websites, and webinars.

The interviews held in the countries concentrated on people's experiences and views and sought to confirm or refute the findings from secondary sources. A total of 36 interviews were performed, most of which involved representatives of vulnerable groups especially pertinent to that country, such as domestic workers and indigenous communities in Brazil and sex workers in India. Interviews were also held with experts, such as health professionals in the DRC, and advocates, such as leaders of social movements in the Philippines.

This paper is not based on comparable datasets and takes into account the real‐life ‘messiness’ of a variety of cases. The comparison is based on what Ludwig Wittgenstein (2009) called ‘family resemblance’ where a series of overlapping similarities connects the cases. Here, the similarities were that the cases fitted some basic characteristics of the low‐intensity conflict in states with authoritarian tendencies and fragile post‐conflict societies that were developed by the When Disaster Meets Conflict programme at the ISS (Hilhorst et al., 2019).

## The cases

Within the first group of cases—that is, low‐intensity or post‐conflict countries—the study of the **DRC** was done by Claire Akello and Christo Gorpudolo. Their report (Gorpudolo and Akello, 2021) focused on the Kivus, a mining region bordering Rwanda. Covid‐19 arrived after more than two decades of protracted violent conflict, compounded by recurrent outbreaks of cholera, Ebola, measles, and Yellow Fever. The poverty level in the DRC is 70 per cent and the health systems are fragile, although there were some coordination capacities for pandemics in relation to Ebola. A drastic national lockdown after the first single instance of Covid‐19 was confirmed wreaked havoc in the two major economic sectors of mining and cross‐border trade.

In **Haiti**, Covid‐19 came to a country that has not experienced war for a long time, but has continuing social conflicts, a notoriously weak governance system, and profound levels of mistrust in authorities and aid actors, exemplified by recurrent protest and political manifestations. Angela Sabogal and Yuki Fujita conducted the research in Haiti (Fujita and Sabogal, 2021).

In the case of **Zimbabwe**, James Kunhiak Muorwel and Lara Vincent examined responses to Covid‐19 amidst political turmoil partly intensified by ongoing economic crises. Despite relatively low Covid‐19 infection rates, the introduction of a total lockdown before a first case was detected had a severe impact on a population highly dependent on the informal economy for their living, a weak health sector, and high levels of corruption, unemployment, and food insecurity (Kunhiak Muorwel and Vincent, 2020; Kunhiak Muorwel, Vincent, and Swartz, 2020).

Within the second group of countries—that is, strong or authoritarian states with a high prevalence of social conflict—Fiorella Macchiavello and Renata Cavalcanti researched the case of **Brazil**. They focused on the struggles of poor urban communities and indigenous peoples in the Amazon to respond to Covid‐19. These groups suffered even more than others as a result of the authoritarian measures of the government and the ensuing complex crisis (Macchiavello and Cavalcanti Muniz, 2021; Macchiavello, Cavalcanti Muniz, and Pegler, 2021).

In the case of **Chile**, the pandemic found a country immersed in a social crisis, with high levels of mistrust in the government, large‐scale social protests, and high levels of unemployment and food insecurity among a vulnerable population. In this context, Ana Isabel Alduenda Avila and Camila Ramos Vilches explored how bottom‐up and communitarian initiatives to respond to Covid‐19 were strongly intertwined with the dynamics of the pre‐existing conflict (Alduenda Avila and Ramos Vilches, 2021; Alduenda and Ramos, 2021).

In **India**, Covid‐19 emerged at a time of intense social protest about the lack of access to official documents by some communities because of their migration history, occupation (and social stigma attached), and overall socioeconomic background. In this setting, Chitrakshi Vashisht and Birendra Singh studied how sex workers, a particularly vulnerable group in the country, struggled with Covid‐19 and the government's measures that ultimately restricted them from working (Singh and Vashisht, 2021a, 2021b).

Lastly, in the **Philippines**, Martin Dacles and Patricia Enriquez explored the impact of Covid‐19 on a country where pockets of conflict continue, mainly in Mindanao, and where state–society relations have been majorly affected by President Rodrigo Duterte's deadly war on drugs. Moreover, an extension of the latter has seen equally deadly measures being deployed against social activists and political opponents (Enriquez and Dacles, 2020, 2021).

## Findings

### Vulnerability and disaster risk creation

The findings from the seven countries confirm that the disaster triggered by Covid‐19 manifested primarily among the most vulnerable segments of the population. Regarding the infection rate of Covid‐19, we do not know what would have happened without the measures taken to curb the pandemic. We do know, however, that they brought about adverse impacts, aggravating enduring economic crises in many cases. Although their benefits in terms of case prevention cannot be gauged, they were also seen to produce new vulnerabilities and thus resulted in disaster risk creation.

In the cases of Chile, the DRC, Haiti, India, and Zimbabwe, while many vulnerable groups were living in a situation of poverty and employed in the informal market, strict lockdowns were imposed and many people saw their source(s) of income and work compromised, resulting in further impoverishment and food insecurity. In Chile, ‘the Covid outbreak was a driving force that worsened conditions already occurring in the country and at the base of the social movement: poverty and informal economy’ (Alduenda Avila and Ramos Vilches, 2021, p. 9).

The worldwide response to Covid‐19 seems to have spilled over to the DRC and Zimbabwe, as the latter announced a state of emergency ahead of the first case, and the initial case in the former triggered a countrywide lockdown. The more deadly Ebola did not lead to such action, and nor did the HIV/AIDS pandemic of yesteryear. The ramifications of the lockdown were severe, with cross‐border trade coming to a standstill and reduced mining activity in the DRC. By the end of April 2020, 62 per cent of the DRC's population feared food insecurity (EurAc, 2020). There were also indications that people's response to the lockdown was adverse in terms of infection risk, with individuals seeking each other's company to cope with the crisis, despite the closures of bars, and reports of women made destitute by the end of their trading activities resorting to transactional sex. In Zimbabwe, the emergency measures also have had far‐reaching repercussions on daily living conditions, including a shortage of *mealie meal* (maize meal), scarcity of fuel, and inflation rate growth of 50 per cent between January and July 2020. ‘As a result, hunger and malnutrition, especially amongst children, spiked, as many cannot provide enough food for themselves and their families’ (Kunhiak Muorwel and Vincent, p. 11). While governments seemingly took responsibility for curbing the pandemic by instigating a state of emergency or lockdown, their subsequent lack of responsibility for the impacts reveals the absence of a social contract in these countries. In the DRC, this is reminiscent of the ‘*débrouillez‐vous*’ or ‘manage yourself’ attitude that has characterised the Congolese government for a long time.

While lockdown was severe in most countries, the effects were uneven, as highlighted by the country reports. The papers focus on vulnerable groups, including the urban poor and indigenous communities, and, in the case of India, the plight of the many sex workers (female, transgender women, and hijras).

### Politics of disaster: instrumentalisation and securitisation

Governments worldwide struggled with responding to Covid‐19, despite continued warnings about the certainty of a pandemic (Moore et al., 2016; Bergeijk, 2021). Without exceptionalising our cases, we observed that ongoing conflict tainted the responses and may have amplified the politics of disaster. What is more, the framing of Covid‐19 as an enemy to defeat was common to a number of the country studies and may have served as a welcome distraction from continuing political and social unrest. The latter was particularly so in Chile with language such as ‘Santiago's battle is the crucial battle in the *war* against coronavirus’ (Alduenda Avila and Ramos Vilches, 2021, p. 11).

In all instances, government responses were instrumentalised for political purposes not related to Covid‐19. This took the form of *over*‐securitisation in a number of cases. The pandemic was used as an excuse to intensify the suppression of social protest, involving the use of excessive force by the police and other units in Chile, Haiti, the Philippines, and Zimbabwe. In Chile, the Philippines, and Zimbabwe, there were reports of arrests and violations of the human rights of members of social protest movements, justified as initiatives to control the virus. In Zimbabwe, authorities used the lockdown ‘to settle scores with opposition supporters and politicians’, selectively arresting opponents for organising small meetings (Kunhiak Muorwel and Vincent, 2020, p. 13). In Haiti, the lockdown was accompanied by strict measures to stop social gathering, effectively ending ongoing social protest in the streets.

Political over‐securitisation could also be accompanied by *under*‐securitisation of the health risks of Covid‐19. Brazil is the most prominent case in point. Covid‐19 responses in the country entailed, on the one hand, a securitisation approach with the adoption of military strategies, bureaucracy, and language, framing the pandemic as an international threat and invisible enemy to be combated (Hoffmann Pfrimer and Barbosa, Jr., 2020). Yet, on the other hand, the country has also became notorious for denying or belittling health risks and displaying an authoritarian sense of control over the pandemic. This under‐securitisation was evident, for instance, in President Jair Bolsonaro's actions to prevent mayors in the country from declaring local lockdowns and blocking other early response actions. President Duterte in the Philippines likewise maintained an image of control for months after the outbreak of the pandemic.

Another form of instrumentalisation was the use of the lockdown and state of emergency to push through controversial and adverse measures. A good example is the Philippines, where the government used the pandemic to pass the Anti‐Terrorism Act of 2020, despite the protest that it had triggered. The fast tracking of the Act was justified as needed in this time of crisis, yet ‘provided the government with the legal tools to oppress and silence those who dissent’ (Enriquez and Dacles, 2020). Another case is Zimbabwe where the resumption of trade was accompanied by the introduction of a registration and tax system. Informal traders could only go back to work if they registered their business, and paid a ‘presumptive tax’, a form of income tax payable by low‐income earners (Parliament of Zimbabwe's Bill Watch 38/2020, in Kunhiak Muorwel and Vincent, 2020, p. 12). The government was thus seen to use the pandemic to increase control over the informal sector.

### Top‐down versus bottom‐up responses

Whereas mismatches between top‐down and bottom‐up responses were perhaps typical of the global response to Covid‐19, this divergence was deepened in a number of our cases by pre‐existing high levels of mistrust in authorities.

The lack of trust in authorities played a prominent role in several of the countries. In Chile, the DRC, Haiti, and Zimbabwe, there was widespread mistrust about the number of Covid‐19 cases, infections, and mortality rates provided by the government. This translated into a lack of legitimacy for the government's actions, complicating the implementation of measures and spawning negative responses, such as denial, resistance, or sabotage. In Haiti, for instance, the ‘disconnection between measures and context was only exasperated by the lack of legitimacy of the government among Haitian people. Therefore, certain communities did not follow the protocols or even rejected them openly’ (Fujita and Sabogal, 2021, p. 19). The governments of Chile and Haiti tried to respond with information and transparency campaigns, but these could not erase the underlying distrust.

As everywhere in the world, people had to rely mainly on their coping mechanisms to deal with the crisis. Initiatives emerged from within communities to help people deal with the health emergency, especially the impact of government measures. Interestingly, in different countries, the mobilisation of social protest that had been taking place before the outbreak transformed into community assistance to respond to the pandemic. Civil society endeavours to address poverty, unemployment, and other issues emanating from conflict or state fragility served as a springboard for Covid‐19 responses.

In Haiti, makeshift voodoo clinics have been an alternative source of healthcare in light of the precarious health system in the country before the pandemic. These have been strengthened and many new ones opened to tackle Covid‐19. In Chile, the *ollas comunes* (common pots), which date back to the dictatorship and have been present since its demise in 1990 in the poorest neighbourhoods as a solution to food insecurity, resurfaced during the social mobilisation and crises of 2018, and once again during Covid‐19. In Brazil, diverse indigenous groups and people living in *favelas* organised themselves to collect food and hygiene items, produce information campaigns, and vocalise their situation and needs. Moreover, gangs in multiple *favelas* imposed curfews to slow the spread of Covid‐19 (Barretto Briso and Phillips, 2020; Soares, 2020). Slum leaders and small‐scale bottom‐up initiatives proved essential in responding to Covid‐19 in India (Auerbach and Thachil, 2021). And community networks and non‐governmental organisations (NGOs) played a role in helping sex workers in India to receive income and food, as their work had become inviable, even though some of them were able to maintain some of it through online or telephone‐based services or were given some support by regular clients.

Lastly, the theme of instrumentalisation reappeared in relation to community donations from large private companies. Similar observations were recorded in several countries that these donations were accompanied by an emphasis on branding (the company name) and media exposure.

## Conclusion

This paper addresses what happened when Covid‐19 met conflict, in a period of six months, from March–August 2020. It focuses on three low‐intensity or post‐conflict countries (the DRC, Haiti, and Zimbabwe) with significant levels of state fragility, and four countries that are characterised as having a strong or authoritarian state and social counter‐movements (Brazil, Chile, India, and the Philippines). The study did not cover high‐intensity conflict scenarios owing, most notably, to a lack of access to data. The choice of countries with low‐intensity conflict and high state fragility makes the paper relevant to many other nations that have similar patterns of social unrest related to fragility or authoritarianism.

The paper builds on analytical frameworks and theories of disaster studies, assessing the cases using a similar set of questions previously posed by the When Disaster Meets Conflict programme of the ISS. An ‘iron law’ of disaster studies dictates that disasters materialise through the interaction of a hazard, vulnerabilities, and responses. As a result, policies, including those meant to mitigate disaster, are often seen to lead to (new) disaster risk creation. While many governments view, or frame, Covid‐19 *as* the disaster, we found that the virus represents a hazard that can turn into a disaster when it interacts with vulnerability, and that it can be exacerbated by actions taken (or not) to address the risk of the pandemic and people's vulnerability. In other words, inadequate responses thus lead to disaster risk creation.

The case studies concentrate in part on vulnerable groups, including indigenous communities, sex workers, and urban poor people living in *favelas* or slums. In all instances, measures taken to address Covid‐19, while reducing the spread of the virus initially, created new risks and accentuated the impact of the pandemic, especially among vulnerable groups. Lockdowns, for instance, affected significantly people working in the informal sector in most of the countries under review, resulting in higher levels of impoverishment and food insecurity. Governments were seen to assume much responsibility by declaring states of emergency and lockdowns, but this was in striking contrast to the lack of responsibility to mitigate the ramifications of the crisis and the measures, pointing to the absence of a social contract as people were left largely to manage by themselves.

Across the case studies, we found instances of disaster politics, whereby Covid‐19 was securitised—either over‐securitised or under‐securitised—and instrumentalised. An *over*‐securitised approach to Covid‐19 entailed strong top‐down response measures such as curfews, quarantines, and restrictions on gathering, which were also used by governments to counter social protest that was prevalent before and during the outbreak and to advance unpopular agendas. These strategies may have been successful in silencing dissident voices, but the root causes of social unrest remained unaddressed or even worsened.

Conditions of social conflict and state–society disarticulation widened the gap between top‐down and bottom‐up initiatives that is often found in disaster responses. First, high levels of mistrust in authorities meant that populations in a number of countries did not believe the figures provided by the government on the pandemic, and the measures lacked legitimacy in their eyes, leading to non‐abeyance or sabotage. Second, and as mentioned above, we did not find instances of a government supporting bottom‐up coping initiatives. Many of the community‐based coping mechanisms were continuities from pre‐Covid times, as the countries were already experiencing crises. Third, a number of community‐based projects were initiated or supported by groups engaged in social protest or activism prior to the outbreak. Where countries targeted social activists, one can assume, therefore, that this also led to governments actively disabling community‐based response mechanisms.

These findings allow us to conclude two main points with regard to the impact of conflict on Covid‐19. These points are consistent with the literature on the nexus of disaster and conflict, as depicted in Figure 1. First, the effects of Covid‐19 as a disaster on people's vulnerability and disaster risk creation are related to previous conflict histories. A disaster like Covid‐19 does not present an easy scenario with which to navigate. Strong top‐down measures are widely considered to be necessary to slow the spread of the virus and to reduce the impacts of the pandemic. However, even though responses to Covid‐19 were seemingly similar at a global level, including lockdowns, the way in which they were given meaning and worked out for affected communities was context‐specific. Social conflict was one of the factors that shaped the reaction to Covid‐19, via the instrumentalisation of the response and varying securitisation tactics. This turned implemented measures into vehicles of disaster risk creation. Second, the handling of a disaster such as Covid‐19 intercedes the effects of conflict and people's vulnerabilities. Whether a disaster will trigger or exacerbate conflict and vulnerabilities depends on pre‐existing, country‐specific conditions and how a government, and other actors, frame and respond to it.

This paper was written on the basis of remote research in the first six month of the pandemic. One of the limitations was that data collection was not uniform across the seven countries. Nonetheless, strong patterns were found with respect to the instrumentalisation of the response and mismatches between top‐down and bottom‐up approaches. How these have worked out, in particular whether or not bottom‐up initiatives to curb the pandemic have been effective in raising support (instead of being oppressed) and mobilising populations to take protective measures, is a matter for subsequent research.

## Acknowledgements

We would like to express great thanks to everyone who worked on this project, especially those who conducted and guided the case studies: Ana Isabel Alduenda, Angela Sabogal, Birendra Singh, Camila Ramos, Chitrakshi Vashisht, Christo Gorpudolo, Claire Akello, Fiorella Macchiavello, Isabelle Desportes, James Kunhiak Muorwel, Lara Vincent, Lee Pegler, Martin Dacles, Patricia Enriquez, Renata Cavalcanti, Roanne van Voorst, Samantha Melis, and Yuki Fujita. This work was supported by the Netherlands Organisation for Scientific Research (grant number: 453‐14‐013) and by the European Research Council under the Horizon 2020 research and innovation programme (grant agreement: 884139).

## Data availability statement

The data that support the findings of this study are available on request from the corresponding author. The data are not publicly available due to privacy or ethical restrictions.
